# Supplementary material for: Cytokine Profiling and Intra-Articular Injection of Autologous Platelet-Rich Plasma in Knee Osteoarthritis
Source: Int J Mol Sci. 2022 Jan 14;23(2):890. doi: 10.3390/ijms23020890 (PMC8779764; doi:10.3390/ijms23020890)
Supplement: Supplementary file 1 [file ijms-23-00890-s001.zip › ijms-1516487-supplementary.pdf]

Table S1. Demographic data of 10 knee OA patients for chondrocyte isolation.

Patients	Age (years)	Gender (F/M)	BMI (kg/m^2)	KL score	VAS (0-10)	KOOS	ISOA	WOMAC
1	72	F	31.20	3	5	25.00	8	147.21
2	69	F	26.64	3	3	25.00	15	115.59
3	65	M	29.05	3	6	43.75	10	253.82
4	77	F	22.06	3	9	12.50	13	202.06
5	77	M	21.33	4	3	12.50	17	52.21
6	65	F	25.24	2	8	12.50	14	121.62
7	73	F	27.34	2	7	12.50	13	160.88
8	67	F	25.68	2	4	37.50	14	201.62
9	81	F	32.65	2	8	37.50	13	155.00
10	72	F	20.08	4	5	25.00	17	105.00

Note: BMI: Body mass index, F: Female, M: Male, KL score: Kellgren and Lawrence grading system, KOOS: Knee injury and Osteoarthritis Outcome Score, ISOA: Indices of severity and disease activity for osteoarthritis, VAS: Visual analog scale, WOMAC: Western Ontario and McMaster Universities Arthritis Index.

Table S2. Primer sequences used for the quantitative real-time PCR analysis.

**Gene Name**	**Forward Primer**	**Reverse Primer**	**Accession Number**
SOX9	5′-ATCTGAAGAAGGAGAGCGAG-3′	5′-TCAGAAGTCTCCCAGAGCTTG-3′	NM_000346.4
COL2A1	5′-CTGGCTCCCAACACTGCCAACGTC-3′	5′-TCCTTTGGGTTTGCAACGGATTGT	NM_001844.5
ACAN	5′-TGAGGAGGGCTGGAACAAGTACC-3′	5′-GGAGGTGGTAATTGCAGGGAACA-3′	NM_001369268.1
GADPH	5′-TTCCATTGACCTCAACTACAT-3′	5′-GAGGGGCCATCCACAGTCTT-3′	NM_002046.7

Note: *SOX9*: SRY-box transcription factor; *COL2A1*: collagen type II alpha 1; *ACAN*: aggrecan; *GADPH*: Glyceraldehyde 3-phosphate dehydrogenase.
